# Authors’ response: Migrant tuberculosis in low-incidence Japan; introduction of pre-entry screening

**DOI:** 10.2807/1560-7917.ES.2025.30.25.2500438

**Published:** 2025-06-26

**Authors:** Gerard de Vries, Sarah Jackson, Brit Häcker, Teresa Domaszewska, on behalf of all co-authors of the original article

**Affiliations:** 1 National Institute for Public Health and the Environment (RIVM), Bilthoven, Netherlands; 2 Health Protection Surveillance Centre, Dublin, Ireland; 3 German Central Committee against Tuberculosis (DZK), Berlin, Germany; 4 Robert Koch Institute, Berlin, Germany; 5 https://www.eurosurveillance.org/content/10.2807/1560-7917.ES.2025.30.11.2400489

**Keywords:** Tuberculosis, low-incidence tuberculosis countries, migrants, cross-border tuberculosis control, tuberculosis screening

**To the editor**: We thank Ujiie and colleagues for sharing information on the recently introduced pre-entry migrant tuberculosis (TB) screening programme in Japan [1]. This initiative responds to the shifting epidemiology of TB in Japan, where individuals born outside of the country now account for 16% of all TB patients [1]. Several European countries have undergone a similar transition; in the 20 low-incidence European countries included in our study, 55% of TB patients were of foreign origin [2].

We presented crude incidence rates (CIRs) of TB among migrant populations using TB surveillance data from The European Surveillance System, managed by the European Centre for Disease Prevention and Control (ECDC), along with population figures from Eurostat. In the countries we examined, the CIRs among migrants from the Philippines, Nepal, and Viet Nam – countries now included in Japan’s pre-entry screening programme – were 67, 148, and 48 per 100,000 population, respectively. Among newly arrived migrants from these countries (within 1 year of arrival), the CIRs were 215, 178, and 85 per 100,000, respectively. It would be of interest to calculate CIRs for these and other migrant populations in Japan.

Furthermore, it would be relevant to understand whether Japan applies additional criteria beyond the proportion of TB cases among the foreign-born individuals with TB when selecting the countries to include in the pre-entry screening programme. For screening, several European countries use thresholds based on the World Health Organization (WHO) estimated TB incidence in the countries of origin and/or other criteria such as actual screening yield in the European destination countries [3,4].

Ujiie et al. also described that together with the increased number of people with TB diagnosed among migrants, TB epidemiology is shifting, with the majority of TB cases in young people being diagnosed among migrants. In our study, we referred to a more detailed recently published analysis of the demographic characteristics of migrant TB patients in our cohort [5]. Patients from the 10 countries with the highest CIRs were younger (median age 25 vs 34 years) and more often male (male-to-female ratio 2.6 vs 1.8) compared with all migrants with TB. The majority (58.1%) of the 114,370 migrants with TB in our cohort originated from countries with a WHO-estimated incidence ≥ 100 per 100,000 population. The highest proportions of people living with HIV (6.7%) and extrapulmonary TB (43.5%) were observed among migrants from countries with a WHO-estimated incidence of ≥ 100 per 100,000. The highest proportions of previous diagnosis (13.7%) and infection with first-line drug-resistant and multidrug-resistant (MDR-TB) strains (12.2% and 6.0%, respectively) were seen among migrants from countries with a WHO-estimated incidence of 40–99.9 per 100,000. Among patients with available data, almost 20% were notified with TB within 2 years after arrival, while 43.1% were diagnosed ≥ 10 years after arrival, highlighting the need for sustained access to TB diagnostic and care services, consistent with the observations made by the Japanese colleagues. In contrast to migrants with TB residing longer in the destination country, migrants notified with TB within 2 years appeared to be younger (median age 28 years vs 49 years in people diagnosed ≥ 10 years post-arrival), with higher proportions of pulmonary (67% vs 52% in people diagnosed ≥ 10 years post-arrival) and MDR-TB (4.4% vs 1.3% in people diagnosed ≥ 10 years post-arrival).

Our two studies, along with a recent scoping review and consensus statement [4,6], highlight several important considerations for TB screening. First, TB disease screening typically targets pulmonary TB, based on symptoms and radiological signs at the time of screening. Consequently, extrapulmonary TB is likely underdiagnosed. Second, this type of screening does not diagnose TB infection and does not prevent infection to disease progression. Therefore, alternative strategies for prevention should be explored for populations at risk. These findings also underscore the importance of raising TB awareness among healthcare providers, ensuring equitable access to TB services for migrant populations and guaranteeing continuity of care for mobile populations. In this respect, in 2024 the ECDC established a working group on TB and Migrant Health, bringing TB experts from 10 European countries together to work on this topic.
